# A Genomic and Epigenetic Comparative Study of Low-grade Biphenotypic Sinonasal Sarcoma with Metachronous and Synchronous High-grade Rhabdomyosarcomatous Transformation

**DOI:** 10.1007/s12105-026-01923-1

**Published:** 2026-05-25

**Authors:** Rayan M. Sibira, Constance Chen, Felipe D’. Almeida Costa, Anuj Verma, Poorva Singh, Jo Elle Peterson, Travis Hattery, Emilian Racila, Minghao Zhong, Paari Murugan, Josephine K. Dermawan

**Affiliations:** 1 Department of Laboratory Medicine and Pathology, University of Minnesota, Minneapolis, MN USA; 2 University of Minnesota Medical School, University of Minnesota, Minneapolis, MN USA; 3 A.C. Camargo Cancer Center, São Paulo, Brazil; 4 DASA Laboratories, São Paulo, Brazil; 5 Department of Pathology, Yale School of Medicine, New Haven, CT USA; 6 Department of Anatomic Pathology, University of Oklahoma Health Sciences Center, Oklahoma City, OK USA; 7 Department of Pathology and Laboratory Medicine, Cleveland Clinic, Cleveland, OH 44195 USA

**Keywords:** Biphenotypic sinonasal sarcoma, *PAX3*, *FOXO1*, *PAX3*, *MAML3*, High-grade rhabdomyosarcomatous transformation

## Abstract

**Background:**

Biphenotypic sinonasal sarcoma (BSNS) is usually histologically distinguishable in its conventional form based on morphology and ancillary studies. However, a subset of BSNS loses characteristic histologic features over time and instead undergoes high-grade rhabdomyosarcomatous transformation (HGRT). The fact that the driver *PAX3* fusion of BSNS overlaps with that of other tumors, e.g., alveolar rhabdomyosarcoma, further complicates this diagnostic dilemma. DNA methylation profiling has emerged as a helpful tool in classifying poorly differentiated malignancies. We sought to investigate whether conventional BSNS and HGRT retain the same epigenetic signature over time.

**Materials and Methods:**

Five pure BSNS samples from the initial diagnostic specimens and 4 HGRT components (3 from the long-term recurrence and 1 synchronous) were collected. An immunohistochemical panel of smooth muscle, skeletal muscle, and neural markers was performed and scored as positive or negative. Molecular analyses included RNA-based (4 cases) and DNA-based (2 cases) next-generation sequencing. DNA methylation profiling using the Illumina Infinium EPICv2 microarray was performed, and the methylomes from paired BSNS and HGRT components were compared with a control cohort of BSNS and various high-grade sarcomas.

**Results:**

The tumors arose from 3 females and one male with an age range of 38–88 years (median 70.5 years old). The time interval between initial BSNS diagnosis to HGRT was between 0 and 14 years (median 8 years). The BSNS components were diffusely positive for S-100 and SMA (4/4), while the HGRT components were positive for desmin (4/4) myogenin (3/4) and Myo D1 (1/1). By RNA sequencing, 2 showed *PAX3::FOXO1* fusion and 2 showed *PAX3::MAML3* fusion. Compared to the low-grade BSNS, the HGRT from the same patients acquired additional secondary genomic alterations and significantly more frequent chromosomal arm-level copy number variations. DNA methylation profiling demonstrated that in 3 patients, both low-grade BSNS and HGRT converged with conventional BSNS. In one patient with *PAX3::FOXO1* fusion, the HGRT from a late recurrence 14 years since initial diagnosis overlapped epigenetically with embryonal rhabdomyosarcoma.

**Conclusion:**

The epigenetic signature of BSNS is usually preserved in cases that underwent HGRT, even after many years from the initial onset of BSNS. However, in rare instances, the HGRT component may display epigenetic divergence over time.

**Supplementary Information:**

The online version contains supplementary material available at 10.1007/s12105-026-01923-1.

## Introduction

Biphenotypic sinonasal sarcoma (BSNS) is a low-grade, slow-growing, non-metastasizing spindle cell neoplasm of the sinonasal tract. Histologically, it is characterized by infiltrative spindle cells arranged in medium to long fascicles with a herringbone pattern, exhibiting minimal atypia, and often showing neuronal and myogenic differentiation [1]. .*PAX3* gene rearrangement is the most commonly observed molecular alteration in BSNS, with various fusion partners including *MAML3, FOXO1, WWTR1, NCOA1, NCOA2,* and *INO80D* [2–5]. However, a minority of cases do not harbor detectable gene rearrangements, and the diagnosis is rendered based on histopathologic and immunohistochemical features [4].

Recent studies have reported high-grade transformation into various spindle cell sarcomas, most commonly fibrosarcoma and rhabdomyosarcoma [6–9]. Epigenetic studies of fusion-positive BSNS cases have shown that BSNS harbors a distinct epigenetic signature, and forms an epigenetic class that is clearly separate from other sinonasal tumors [10].

In this study, we present four unique cases of BSNS associated with high-grade rhabdomyosarcomatous (or rhabdomyoblastic) differentiation (HGRT): three late recurrence, one synchronous (including two previously reported cases without methylation data), [7, 9],to evaluate the epigenetic evolution of low-grade BSNS as it undergoes HGRT by comparing the methylomes of the original low-grade tumors with their corresponding transformed high-grade components.

## Materials and Methods

### Study Cohort

The study cohort include two previously published cases of BSNS with *PAX3::MAML3* (including both low-grade and high-grade components) provided by coauthors (J.P, A.V) [7, 9]. Additionally, our archives were searched for cases of BSNS with high-grade transformation: two cases *PAX3::FOXO1* were provided by the authors (R.S, E.R, M.Z, P.M). Clinical data—including age, sex, anatomic location, radiologic findings, treatment details, and recurrence information—were obtained through clinical chart reviews. Hematoxylin and eosin–stained slides from biopsy, excision, and resection specimens, were re-evaluated. Five pure BSNS samples from the initial diagnostic specimens and four HGRT components samples (three from the long-term recurrence and one synchronous) were collected. In patient 3, additional immunohistochemical profile and follow-up information have been updated, while in patient 4, RNA sequencing was done to clarify the *PAX3* fusion partner (Table 1). The study was approved by the Institutional Review Board of the participating institutions.

**Table 1:** Clinicopathologic features of studied biphenotypic sinonasal sarcoma patients with high grade rhabdomyosarcomatous transformation

Patient no	Age at diagnosis	Sex	Location	Primary size (cm)	HGRT size (cm)	Follow-up	Outcome	Positive immunohistochemical markers		*PAX3* partner
								Low grade	High grade	
1	38	F	Frontal and anterior left ethmoid air cells sinuses	5.6	2.8	14 years	DOD	SMA, S-100	Desmin, myogenin, Myo D1	*FOXO1*
2	69	F	Right superior nasal cavity	4.7	4.4	8.5 years	DOD	SMA, S-100	Desmin, myogenin	*FOXO1*
3	88	M	Left nasal cavity, superior meatus	9.1	6.4	8 years	DOD	SMA, S-100	Desmin, S-100 (f)	*MAML3*
4	72	F	Entire sinonasal cavities bilaterally	–	–	4.5 months	DOD/DOO	SMA, S-100, Desmin (f), myogenin (f)	Desmin, myogenin	*MAML3*

DOD, died of disease; DOO, died of others; f, focal

### Immunohistochemistry

The relevant immunohistochemical antibodies and clones used in this study were as follows: smooth muscle actin (clone 1A4, mouse monoclonal antibody), S-100 (rabbit polyclonal antibody), desmin (clone DE-R-11, mouse monoclonal antibody), myogenin (clone FD5, mouse monoclonal antibody) and Myo D1 (clone EP212, rabbit monoclonal antibody). Immunostains were performed with appropriate positive and negative controls.

### Targeted RNA Sequencing

An RNA-based next-generation sequencing (NGS) assay—NGS Oncology (NGSO) Pan-Tumor Fusion Assay—was used, which is validated for the detection of a wide spectrum of oncogenic fusions in solid tumors, including sarcomas. The assay comprehensively targets 127 genes of established significance in a partner-agnostic fashion and utilizes anchored multiplex PCR (AMP) chemistry to enable detection of both known and novel fusion partners. Total nucleic acid is extracted using the Promega Maxwell Instrument with the Maxwell RSC FFPE Kit and quantified using a Qubit 2.0 Fluorometer. Library preparation is performed according to the manufacturer’s protocol and sequenced on an Illumina instrument. The generated FASTQ files are processed using ArcherTM analysis pipeline for fusion calling.

### DNA Molecular Profiling

In patient 1 and 2, genomic DNA was extracted from the sample, and sequencing libraries were prepared using a custom-designed hybrid capture–based assay (Integrated DNA Technologies). The enriched DNA libraries were sequenced on an Illumina MiSeq or NextSeq 550 instrument, and the resulting FASTQ files were processed through the GenomOncology a custom-developed bioinformatics pipeline to call sequence variants (single nucleotide variants and insertion–deletion variants), copy number alterations (amplifications and deletions), and to calculate tumor mutation burden (TMB) and Microsatellite Instability (MSI). Mutations and gene-level copy number alterations were visualized and summarized as an oncoprint using the R package “ComplexHeatmap” version 2.8.0.

### DNA Methylation Profiling

Methylation profiling was performed on both the low-grade and high-grade components of four cases of BSNS with *PAX3::FOXO1* (patient 1 and patient 2) and *PAX3::MAML3* (patient 3 and patient 4) fusions, respectively. Details of DNA methylation profiling have been published previously [11]. In brief, genomic DNA was extracted from either formalin-fixed, paraffin-embedded (FFPE) curls (submitted in 1.5 or 2.0 mL DNase/RNase-free microcentrifuge tubes) or FFPE tissue sections for each sample. For DNA extraction from FFPE curls, Qiagen’s QIAamp DNA FFPE Tissue Kit (Cat. No./ID: 56404) was used, following the manufacturer’s instructions. Next, 100–250 ng of genomic DNA was subjected to bisulfite conversion and processed on the Illumina methylation EPIC v2.0platform according to the manufacturer’s instructions.

For comparison, raw IDAT files derived from Illumina methylation 450 k or EPICv1 platforms for small round blue cell tumors were obtained from the Heidelberg sarcoma methylation classifier reference cohort (Gene Expression Omnibus study accession number GSE140686). Methylome profiles included 36 alveolar rhabdomyosarcomas (ARMS), 8 round cell tumors with *BCOR* alterations (BCOR), 11 small round blue cell tumors with *CIC* alterations (CIC), 26 desmoplastic small round cell tumors, 30 embryonal rhabdomyosarcomas (ERMS), and 37 Ewing sarcomas. Additionally, IDAT files from 37 cases of ARMS with confirmed *FOXO1* rearrangements by FISH (11 *PAX3::FOXO1* cases, 3 *PAX7::FOXO1* cases, and 23 cases with unknown 5′ partners) were downloaded from GSE167059 [12].

Furthermore, IDAT files from two previously published cases of ARMS with *PAX3::NCOA1* and *PAX3::INO80D*, respectively, one unpublished case of ARMS with *PAX3::NCOA1*, and two previously published cases of ARMS with *PAX3::MAML3* were kindly provided by author J.D [11]. Additionally, IDAT files from 11 cases of BSNS with *PAX3::MAML3* (7 cases), *PAX3::NCOA2* (1 case), *PAX3::FOXO1* (2 cases), and *PAX3::YAP1* (1 case) were kindly provided by coauthor F.C.

### Methylation Data Analysis

IDAT processing and data analysis on all samples was performed using R version 4.2.0 and the “minfi” package. Normalization was performed using the preprocess Illumina function and probes with a detection *P* value > 0.01 were filtered, as were SNP-related probes, and probes on sex chromosomes. After probe filtering and intersecting internal and external datasets, 230,345 CpG probes remained for downstream analysis. Methylation levels were measured using beta values (ratio of the methylated probe intensity to the overall intensity—sum of methylated and unmethylated probe intensities) for all cases.

The top 25,000 most variable CpGs by variance were analyzed by principal component analysis (PCA) (prcomp function). The top principal components (PCs) that explained at least 80% of cumulative variance were input into umap2 function (uwot R package 0.2.2) for uniform manifold approximation and projection (UMAP) analysis, setting random seed to 123, number of neighbors as 5, min_dist to 0.1 and metric to euclidean. Additionally, unsupervised hierarchical clustering was performed on the normalized beta value data matrix using the top 5000 most variable CpGs by variance using the heatmap R package version 1.0.12 with Ward.D2’s linkage and Euclidean distance for clustering.

For copy number variation (CNV) analysis, the Mset files after IDAT files preprocessing were processed by “conumee2” R package version 2.1.2. For normal controls, a set of 20 normal blood samples from GSE286313 processed by the same EPICv2 platform were downloaded. Both sets of idat files were processed by “preprocessIllumina” function of the minfi package. The conumee2 annotation object was created using “CNV.create.anno” function form conumee2 package setting array_type to “EPICv2”. The experimental and control data are loaded with “CNV.load” function, followed by CNV calling with “CNV.fit”, “CNV.bin”, and “CNV.segment” functions. The CNV.segment output includes chromosomes, start, end, num.mark and segmental mean information. For visualization of summary level CNV, “CNV.summmaryplot” function from “conumee2” was used. For visualization of case-level CNV segments, overlapping segments were derived using the “CNTools” package version 1.52.0 with “CNSeg” followed by “getRS” function. The average copy number signals were then calculated at 1 MB genomic windows and visualized using the R package “ComplexHeatmap” version 2.8.0. CNV was considered present if the absolute segmentation mean is greater or equal to 0.2. Fraction of CNV across the genome was calculated by dividing the sum of all segments with CNV by the sum of all segments across the genome.

## Results

### Clinical Presentation, Management and Follow up

**Fig. 1:**
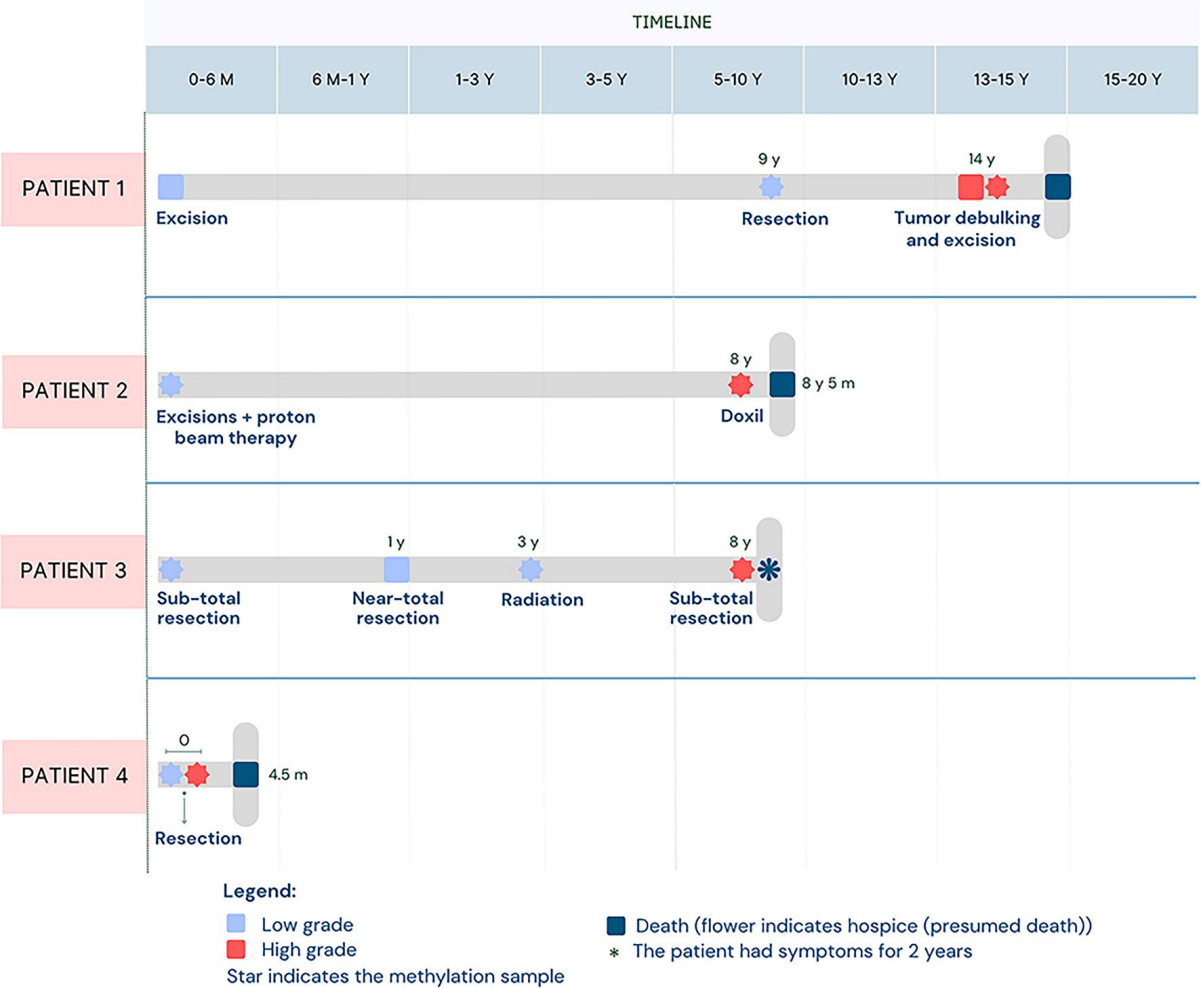
Schematic representation of the clinical timelines and management strategies for the four patients with biphenotypic sinonasal sarcoma with high-grade rhabdomyosarcomatous transformation

**Fig. 2:**
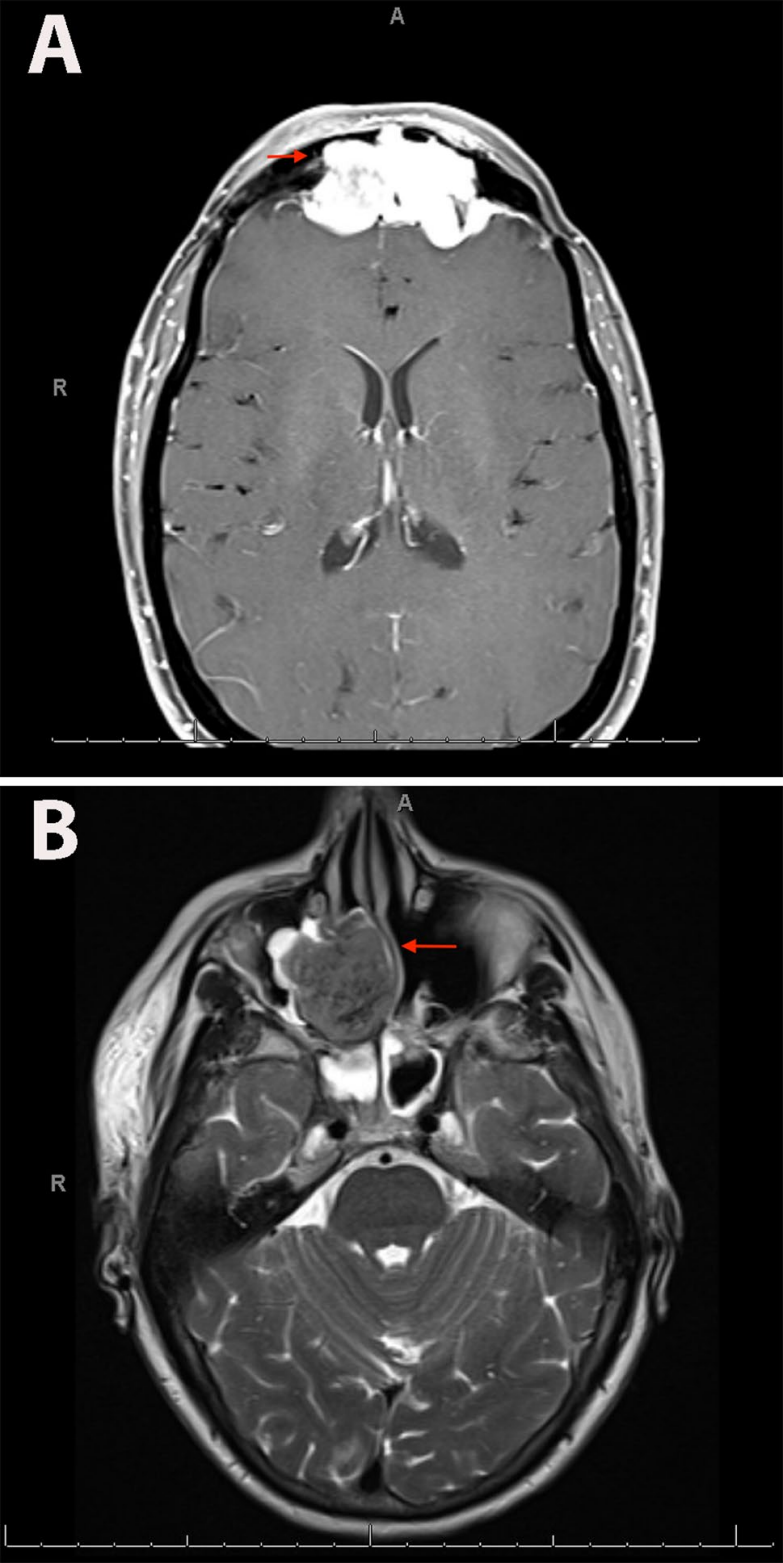
Biphenotypic sinonasal sarcoma, radiologic findings. **A** Patient one: MR orbit, face, and neck (T1-weighted, axial, post-contrast phase) shows a 5.6 cm enhancing mass centered in the frontal sinuses, with thickened, enhancing dura superior to the mass. **B** Patient two: MR brain (T2-weighted, axial) shows a 4.7 cm right paramedian sinonasal mass that obliterates the ethmoid air cells and the right nasal cavity and extends into the right maxillary sinus

Four cases of BSNS with high-grade rhabdomyosarcomatous transformation (HGRT) were identified, including two previously published cases (patient 3 and 4). The cohort consisted of three females and one male, with ages at diagnosis ranging from 38 to 88 years (median, 70.5 years). The interval from initial BSNS diagnosis to development of HGRT ranged from 0 to 14 years (median, 8 years): patients 1–3 exhibited HGRT as a late recurrence, while patient 4 presented with synchronous low-grade BSNS and HGRT in the same resection. Patient 1 was treated with excision of the primary tumor and developed a low-grade recurrence 9 years later, followed by two HGRT recurrences at 14 years, all of which were treated with excision. Patient 2 was treated for the primary tumor with multiple excisions and proton beam therapy and developed an HGRT recurrence 8 years later, which was treated with Doxil chemotherapy. Patient 3 was initially treated with subtotal resection and developed recurrences at 1 and 3 years, which were treated with near-total resection and radiation therapy, respectively; 8 years later, he developed a third recurrence with HGRT that was treated with subtotal resection [9]. In patient 4, the lesion was initially biopsied and subsequently treated with surgical resection [7]. All patients passed away shortly after the diagnosis of HGRT. A clinicopathologic summary is provided in Table 1, and a schematic representation of the clinical timelines, management strategies and outcomes for all four patients is illustrated in Fig. 1. Radiologic findings are shown in Fig. 2.

### Histopathologic and Immunohistochemical Findings

#### Low-grade Biphenotypic Sinonasal Sarcoma (BSNS)

**Fig. 3:**
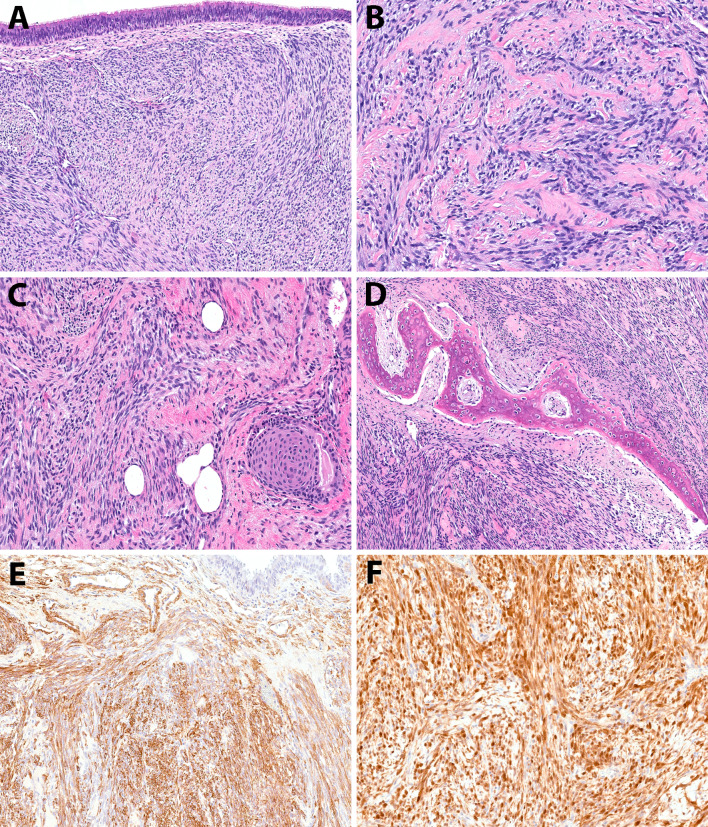
Low grade biphenotypic sinonasal sarcoma. **A** Low-grade spindle cell proliferation in a herringbone pattern beneath the respiratory mucosa, interspersed with thin-walled blood vessels (patient one). **B** Intercellular collagen between spindle cells (patient one). **C** Low-grade spindle cells with squamous metaplasia of the respiratory epithelium (patient two). **D** Entrapped bone within the tumor (patient two). **E** Tumor cells showing diffuse positivity for SMA IHC (patient one).** F**. Tumor cells positive for S-100 IHC (patient two)

The morphologic features of the studied tumors were generally similar. Histologic examination of the primary excision/resection specimens in all patients, as well as the low-grade recurrence specimens (Fig. 3A–D), revealed a spindle cell proliferation undermining invaginated respiratory mucosa. The tumor exhibited an infiltrative growth pattern composed of cells arranged in medium to long fascicles with a herringbone architecture, interspersed with thin-walled blood vessels. The tumor cells contain elongated or wavy nuclei and scant amphophilic cytoplasm. Hypocellular areas with delicate intercellular collagen strands were observed between the cells. Mitotic activity was low (< 1 per 20 high power fields), and there was no evidence of necrosis, surface mucosal ulceration, or hemorrhage. Immunohistochemical (IHC) staining (Fig. 3E, F) showed that the tumor cells were diffusely positive for S-100 and smooth muscle actin (SMA) (4/4). In patient 4 desmin and myogenin were focally positive. The morphology and IHC findings were consistent with BSNS.

### High-grade Rhabdomyosarcomatous (or Rhabdomyoblastic) Transformation (HGRT)

**Fig. 4:**
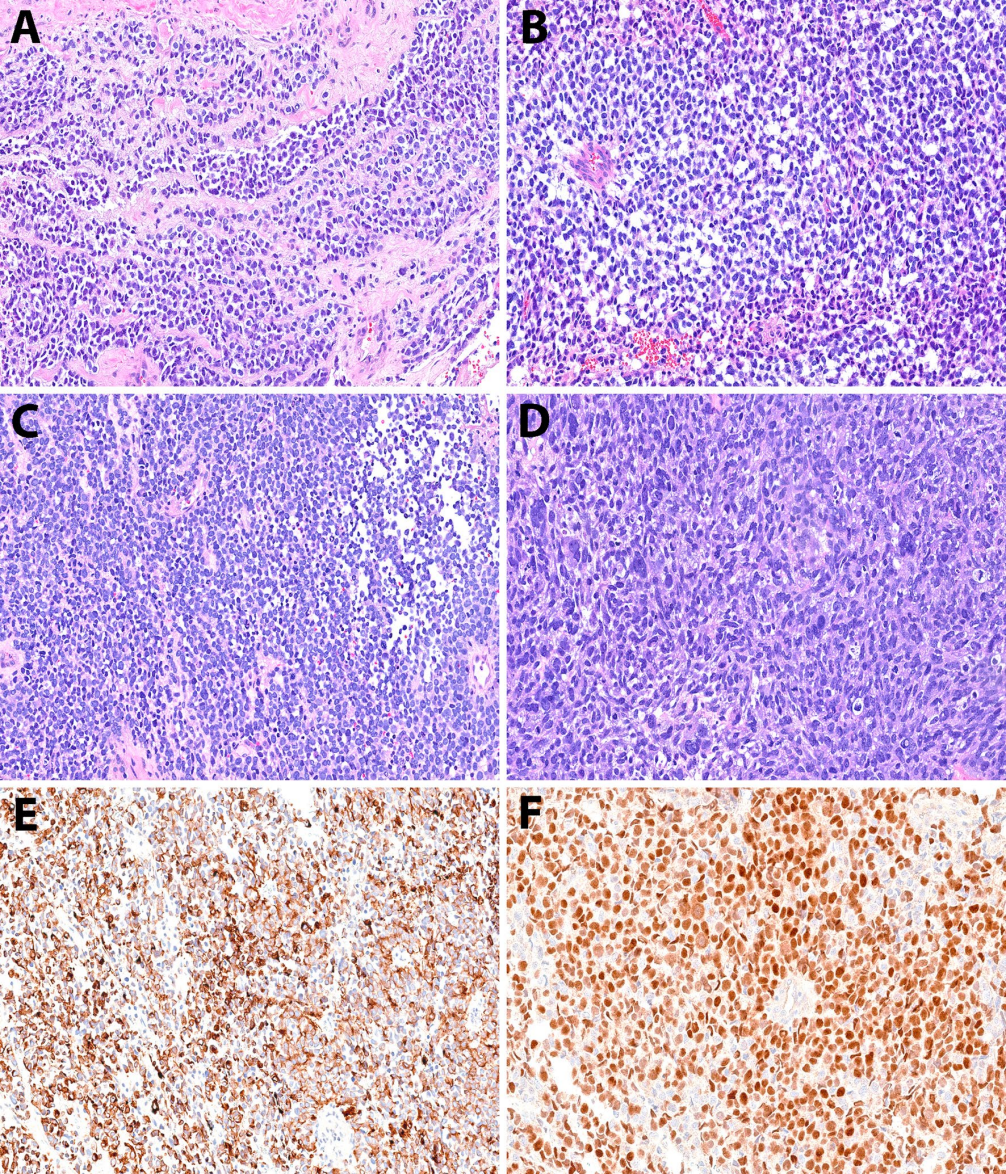
High-grade rhabdomyosarcomatous transformation. **A**–**B** Rhabdomyoblastic transformation with sheet-like and infiltrative growth of high-grade primitive cells (patient one). **C** Patient two. **D** Bizarre spindle cells with brisk mitotic activity (patient two). **E** Rhabdomyoblastic cells are positive for desmin IHC (patient two). **F** Rhabdomyoblastic cells are positive for myogenin IHC (patient one)

Histologic examination of the HGRT component (Fig. 4A–D) showed high-grade, diffuse, and infiltrative tumor growth. The tumor cells demonstrated areas of bizarre nuclear atypia, along with prominent regions composed of sheets of primitive and relatively uniform cells ranging from round to spindly, with scant eosinophilic cytoplasm. In some cases, areas with a fibrosarcomatous growth pattern were also observed. Mitotic activity was brisk, and large areas of necrosis were present. IHC staining (Fig. 4E, F), demonstrated that the tumor cells were positive for desmin (4/4) myogenin (3/4), and Myo D1 ((1/1), diffuse) and negative for SMA (4/4). In patient 3, S-100 was focally positive. These features were consistent with recurrent BSNS with HGRT (de novo in patient 4).

### Molecular Genetic Findings

#### PAX3::FOXO1 Fusion

Targeted RNA sequencing revealed in-frame gene fusion transcripts in patients 1 and 2. In both patients (and in both tumor components), the fusion involved exon 7 of *PAX3* (NM_001127366.3) and exon 2 of *FOXO1* (NM_002015.4) (breakpoint: chr2:223,084,859::chr13:411,349,97).

#### PAX3::MAML3 Fusion

For patient 3, molecular studies of the original tumor demonstrated *PAX3::MAML3* fusion [9]. For patient 4, targeted RNA sequencing confirmed an in-frame *PAX3::MAML3* fusion involving exon 7 of *PAX3* (NM_181459.4) and exon 2 of *MAML3* (NM_018717.5) (breakpoint: chr2:223,084,859::chr4:140,812,121).

### Distinct Somatic Molecular Features in Tested Cases

**Fig. 5:**
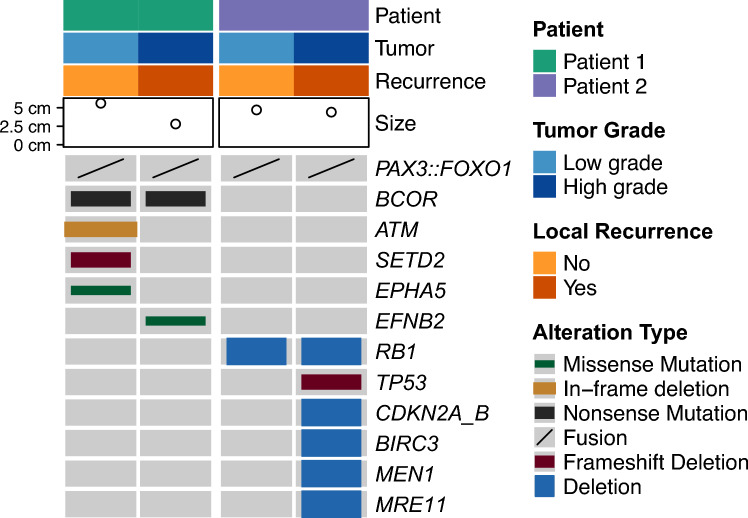
Genomic profile of biphenotypic sinonasal sarcomas with *PAX3::FOXO1* fusion. Oncoprint depicting age, tumor size, tumor grade, local recurrence, frequency, and types of pathogenic variants, as well as alteration types

DNA sequencing was performed on separate specimens of low-grade BSNS and subsequent high-grade recurrences from two cases (patients 1 and 2) with *PAX3::FOXO1* fusion. In patient 1, the low-grade component harbored a *BCOR* nonsense mutation, an *ATM* in-frame deletion, a *SETD2* frameshift deletion, and an *EPHA5* missense mutation. The HGRT retained the *BCOR* nonsense mutation and acquired an additional *EFNB2* missense mutation. In patient 2, the low-grade component showed an *RB1* deletion, and the HGRT retained the *RB1* deletion and acquired a *TP53* frameshift deletion as well as broad deletions of *CDKN2A/B, BIRC3, MEN1,* and *MRE11*. Figure 5 summarizes the genomic profiles of paired samples from patients 1 and 2.

### Unsupervised Clustering Analysis of DNA Methylation

**Fig. 6:**
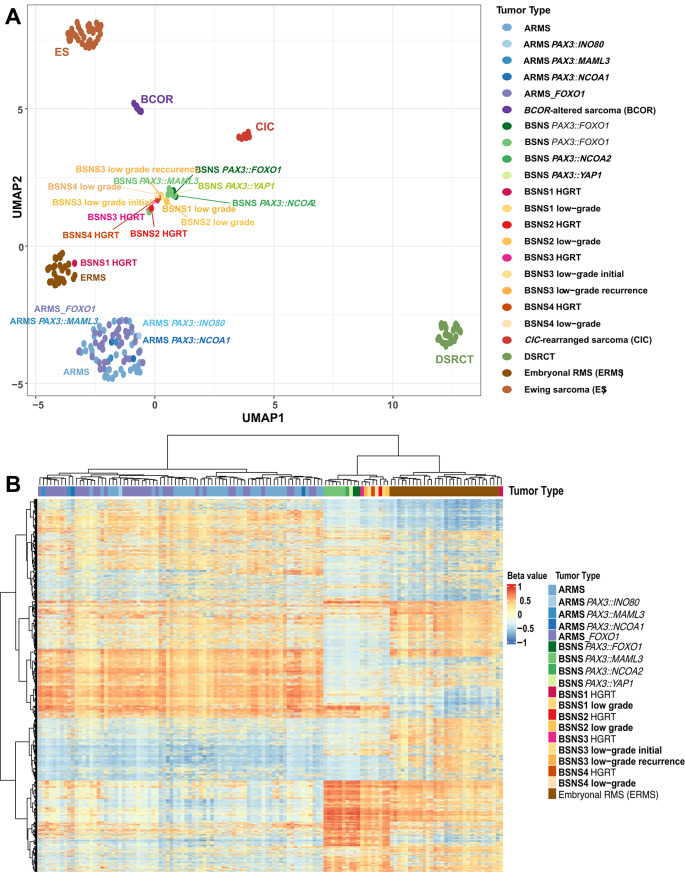
DNA methylation analysis. **A** Dimensionality reduction analysis by uniform manifold approximation and projection (UMAP) of DNA methylation profiles of low-grade BSNS and their high-grade rhabdomyosarcomatous transformation (HGRT) components. Reference cohorts include BSNS with *PAX3* fusions (*PAX3::FOXO1*, *PAX3::MAML3*, *PAX3::YAP1*, *PAX3::NCOA2*) and other sarcoma methylation classes, including embryonal rhabdomyosarcoma (ERMS), alveolar rhabdomyosarcoma (ARMS; including *PAX3::INO80*, *PAX3::MAML3*, *PAX3::NCOA1*, and *FOXO1*-rearranged subtypes), *CIC*-rearranged sarcoma, *BCOR*-altered sarcoma, Ewing sarcoma (ES), and desmoplastic small round cell tumor (DSRCT). Low-grade BSNS and HGRT samples from patients 2–4 converged with the reference BSNS, whereas in patient 1 the low-grade BSNS converged with the reference BSNS and the HGRT component overlapped with ERMS. **B** Heatmap displaying unsupervised hierarchical clustering across the same cohort confirms clustering of low-grade BSNS and most HGRT components (patients 2–4) with the BSNS methylation class. In patient 1 the low-grade component clusters with BSNS and the HGRT component clusters with ERMS

Unsupervised analysis of genome-wide DNA methylation patterns was performed to compare the methylation profiles of the four cases of BSNS with *PAX3::FOXO1* and *PAX3::MAML3* (including both low- and high-grade components), with those of ARMS, BSNS, ERMS, and other small round blue cell tumors. By dimensionality reduction analysis with Uniform Manifold Approximation and Projection (UMAP) and unsupervised hierarchical clustering (Fig. 6A, B), the methylomes of the low-grade BSNS cases overlapped with all other BSNS irrespective of the fusion variant. Among the cases with HGRT, two *PAX3::MAML3*-HGRT cases and one *PAX3::FOXO1*-HGRT case demonstrated epigenetic convergence with low-grade BSNS. In contrast, while the low-grade BSNS tumor with *PAX3::FOXO1* from patient 1 converged with conventional BSNS, the corresponding HGRT from the second recurrence 5 years later overlapped with ERMS. Notably, this case harbored a *BCOR* nonsense mutation in both the BSNS and HGRT components.

### Copy Number Analysis

**Fig. 7:**
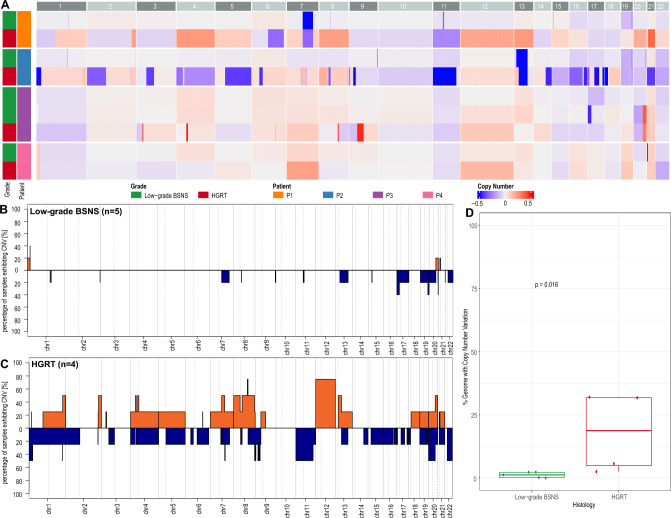
Genomic plots comparing copy-number variation (CNV) profiles between low-grade BSNS and HGRT samples in all four patients. **A** CNV heatmap across all paired samples by patients. **B** Frequency of CNVs by chromosome among low-grade BSNS cases. **C** Frequency of CNVs by chromosome among HGRT cases. **D** Boxplots depicting distribution of percentage of the genome with CNV between the two groups (student t-test)

Next, leveraging probe coverage across the genome on the Infinium methylation array, copy number variation (CNV) analysis was performed against a set of reference normal blood samples. Whole-genome copy number profiles derived from DNA methylation profiling showed fewer CNVs in low-grade BSNS and more frequent, mostly non-recurrent chromosomal arm-level copy-number gains and losses in HGRT samples, which also showed a significantly higher percentage of genome involved by copy number variation (CNV) on average *(*t-test *p* = *0.016)* (Fig. 7 A-D). In patient 1, copy-number variation (CNV) analysis of the low-grade BSNS component showed focal deep chromosomal arm-level deletion on 7q, whereas HGRT showed multiple copy-number gains on 1q, 4–5, 7p, 8, 12, 13, 20–21 and losses on 6q, 7q and 11 (Supplementary material 1). In patient 2, low-grade BSNS CNV analysis showed focal deep chromosomal arm-level deletions on chr13q, which contains *RB1* at 13q14.2, whereas the corresponding HGRT also showed frequent copy-number gains and losses, including chromosomal arm-level deletions on 1p, 2p, 3p, 4q, 5q, chr9p (contains *CDKN2A/CDKN2B* at 9p21.3), 11q (contains *MEN1* at 11q13, *MRE11* at 11q21 and *BIRC3* at 11q22.2), 13q, 17p (includes *TP53* at 17p13.1), and 21 (Supplementary material 1).

### Discussion

Biphenotypic sinonasal sarcoma (BSNS) is characterized by a low-grade infiltrative mesenchymal neoplasm with dual neural and myogenic differentiation, most often harboring *PAX3* rearrangement or other related fusions. BSNS is generally indolent, however, over time some cases show high-grade transformation, raising questions about biological evolution. Although upon high grade transformation the fusion gene remains constant, clinical behavior and morphology change dramatically. We hypothesize that epigenetic reprogramming may be a driving mechanism in this transformation.

DNA methylation is stable, reproducible, and useful in tumor classification. It is a technique that identifies patterns of methylcytosine groups on DNA, a type of epigenetic marker that reflects cell type and tumor evolution. It has been used to refine the classification of high-grade sinonasal tumors, revealed distinct molecular entities for sinonasal undifferentiated carcinomas, and provided a reliable molecular tool for diagnosis and prognosis stratification [13, 14]. A recent study conducted by Costa et al. analyzed fourteen cases of BSNS using DNA methylation profiling and RNA sequencing to investigate whether fusion type or high-grade transformation affects the tumor’s molecular identity. Despite diverse gene fusions, BSNS cases consistently converged together as a distinct epigenetic group, separate from other sinonasal and soft tissue tumors. This finding was unaffected by tumor grade; two tumors with *PAX3::MAML3* and *FUS::POU2AF3* showed synchronous and metachronous high-grade transformation, respectively [10].

We investigated cases of BSNS with high-grade transformation to identify temporal epigenetic alterations (1 synchronous, 3 metachronous/late recurrence). Upon transformation, BSNS frequently exhibits focal or diffuse rhabdomyosarcomatous differentiation, as observed in the present study, including in cases harboring a *PAX3::FOXO1* fusion. On the other hand, a recent study by Dermawan et al. identified the *PAX3::MAML3* fusion in two cases of ARMS that were epigenetically distinct from BSNS [11]. This illustrates the utility of epigenetic profiling to distinguish genetically overlapping tumor types. Additionally, the detection of a non-*FOXO1* rearrangement in ARMS carries significant clinical implications, as current therapeutic protocols rely heavily on *FOXO1* status for risk stratification and treatment decisions in pediatric rhabdomyosarcomas [15]. While adjuvant radiation therapy (photon or proton beam) remains the standard approach for most sinonasal tumors—particularly in patients with advanced T-stage, bony or neural invasion, dural or intracranial involvement, positive margins, or tumors not amenable to complete resection due to high surgical morbidity [16], there are currently no established guidelines for the management of low-grade BSNS or BSNS with high-grade transformation.

To better understand the biology of HGRT in BSNS, we performed DNA methylation profiling on both the low- and high-grade components and compared them with other predominantly low-grade BSNS, fusion-positive and fusion-negative ARMS, ERMS, and other small round blue cell tumors. Comparison of methylomes revealed that both components of the *PAX3::MAML3* BSNS cases, as well as one *PAX3::FOXO1* case, converged with conventional BSNS. However, one *PAX3::FOXO1*-positive BSNS case demonstrated divergent DNA methylome over time: while the low-grade component converged with conventional low-grade BSNS, the high-grade component overlapped with ERMS. Although our findings largely align with the previous study on BSNS by Costa et al. [10], the single case of high-grade BSNS with *PAX3::FOXO1* rearrangement that demonstrates epigenetic shift over time warrants further investigation to determine whether this phenomenon is unique to *PAX3::FOXO1*-associated BSNS or may also occur in other tumor types over the course of oncogenic transformation. Alternatively, what we observed could represent selective overgrowth of a clone within a tumor that already exhibited intratumoral methylation heterogeneity. Elucidating the underlying pathogenesis would likely require advanced techniques, such as single-cell methylation profiling, in future studies.

Interestingly, for the aforementioned case, molecular testing of both components revealed a pathogenic *BCOR* mutation. *BCOR* alterations have been observed predominantly in conventional ERMS [17]. This finding raises the possibility that this case acquired an ERMS-like epigenetic profile upon transformation, possibly due to *BCOR* alterations. However, this needs to be further elucidated across both low- and high-grade BSNS, as well as in cases harboring *PAX3::FOXO1*.

With regard to the variants identified in the low-grade BSNS component that were not detected in the HGRT component, *ATM* and *SETD2* mutations had relatively low variant allele frequencies (VAFs) (6% and 25%, respectively), compared with the *BCOR* variant (VAF of 86%). This may be attributed to technical variations in sensitivity between different sequencing attempts. Another possibility is intratumoral heterogeneity and clonal divergence, whereby a subclone that harbors only a subset of the variants (ie, *BCOR*) subsequently recurs and acquires additional genomic alterations during the course of oncogenic transformation.

The *PAX3::FOXO1* fusion results from the chromosomal translocation t(2;13)(q35;q14), commonly observed in ARMS. In this fusion, the N-terminal portion of *PAX3*—comprising the paired box domain (exons 2–4) and the homeobox domain (exons 5–7)—is fused in-frame with the C-terminal region of *FOXO1*, which includes the forkhead DNA-binding domain and a strong transactivation domain (typically exons 2–7) [18, 19]. The *PAX3::MAML3* fusion arises from the chromosomal rearrangement t(2;4)(q35;q31). Similar to *PAX3::FOXO1*, it retains the N-terminal DNA-binding domains of *PAX3* (paired box domain, exons 2–4; homeobox domain, exons 5–7) fused to the robust transcriptional activation domain of *MAML3* (exon 2 onward) [2, 4, 11, 20]. The chimeric protein in both fusions acts as an aberrant transcription factor, inducing significant chromatin remodeling and lineage shifts, which presumably leads to the dual myogenic and neural differentiation observed in low-grade BSNS [2, 4, 20]. Upon high-grade transformation, the methylome of BSNS typically remains stable, suggesting epigenetic and biological similarity over time. However, in rare cases, epigenetic and biological divergence may occur.

In conclusion, this study comprehensively investigated biphenotypic sinonasal sarcoma (BSNS) from multiple angles. It included both short- and long-term clinical follow-up of patients and performed longitudinal methylation profiling across low-grade and synchronous/metachronous high-grade rhabdomyosarcomatous components. The methylomes of BSNS with *MAML3* or *FOXO1* rearrangements were compared to those of fusion-positive and fusion-negative rhabdomyosarcomas, revealing epigenetic patterns in high-grade HGRT that are consistent with conventional BSNS (Patient 2, 3, and 4). These findings build upon and consolidate previous work (Costa et al. [10]), supporting the utility of DNA methylation profiling in prognostication and therapeutic stratification, particularly in cases of BSNS with HGRT. However, one limitation is that, aside from BSNS cases, clustering analyses relied on publicly available methylation datasets, which may differ in quality, platform, and data preprocessing pipelines. While the study successfully distinguished high-grade BSNS with HGRT from ARMS and ERMS in 3 of the patients, the distinct methylation profile observed in the HGRT component of patient 1 (with *PAX3::FOXO1* fusion) warrants further investigation. It remains unclear whether this finding represents a coincidental epigenetic divergence or a phenomenon that could be observed in additional cases. Clarifying this may have implications for biological classification and therapeutic decision-making in future studies.

## Supplementary Information

Below is the link to the electronic supplementary material.

Supplementary file1 (DOCX 13 KB)

Supplementary file2 (PDF 34913 KB)

## Data Availability

The data generated during the current study are not publicly available due to patient confidentiality but are available from the corresponding author on reasonable request.
