# Supplementary material for: A Genomic and Epigenetic Comparative Study of Low-grade Biphenotypic Sinonasal Sarcoma with Metachronous and Synchronous High-grade Rhabdomyosarcomatous Transformation
Source: Head Neck Pathol. 2026 May 25;20(1):49. doi: 10.1007/s12105-026-01923-1 (PMC13201838; doi:10.1007/s12105-026-01923-1)
Supplement: Supplementary file 1 — Supplementary file1 (DOCX 13 KB) [file 12105_2026_1923_MOESM1_ESM.docx]

**Supplementary material 1:**

Paired copy-number variation (CNV) profiles of patient 1 and 2 with *PAX3::FOXO1* comparing LG-BSNS and HGRT.
