# Supplementary figures and images for: A Genomic and Epigenetic Comparative Study of Low-grade Biphenotypic Sinonasal Sarcoma with Metachronous and Synchronous High-grade Rhabdomyosarcomatous Transformation
Source: Head Neck Pathol. 2026 May 25;20(1):49. doi: 10.1007/s12105-026-01923-1 (PMC13201838; doi:10.1007/s12105-026-01923-1)

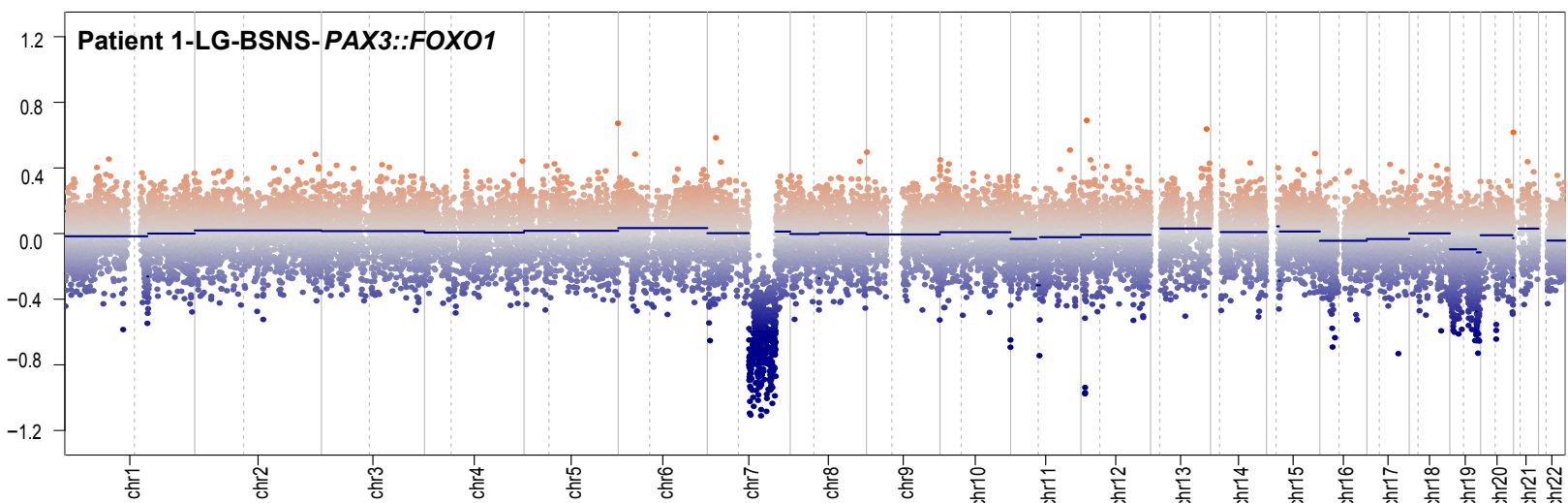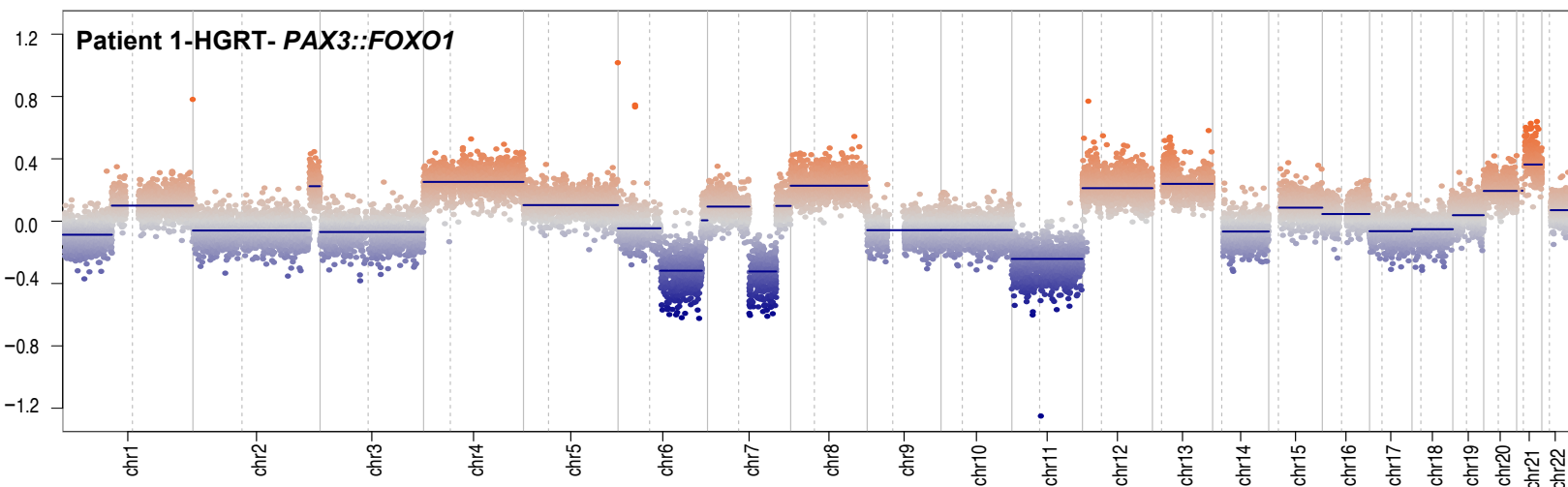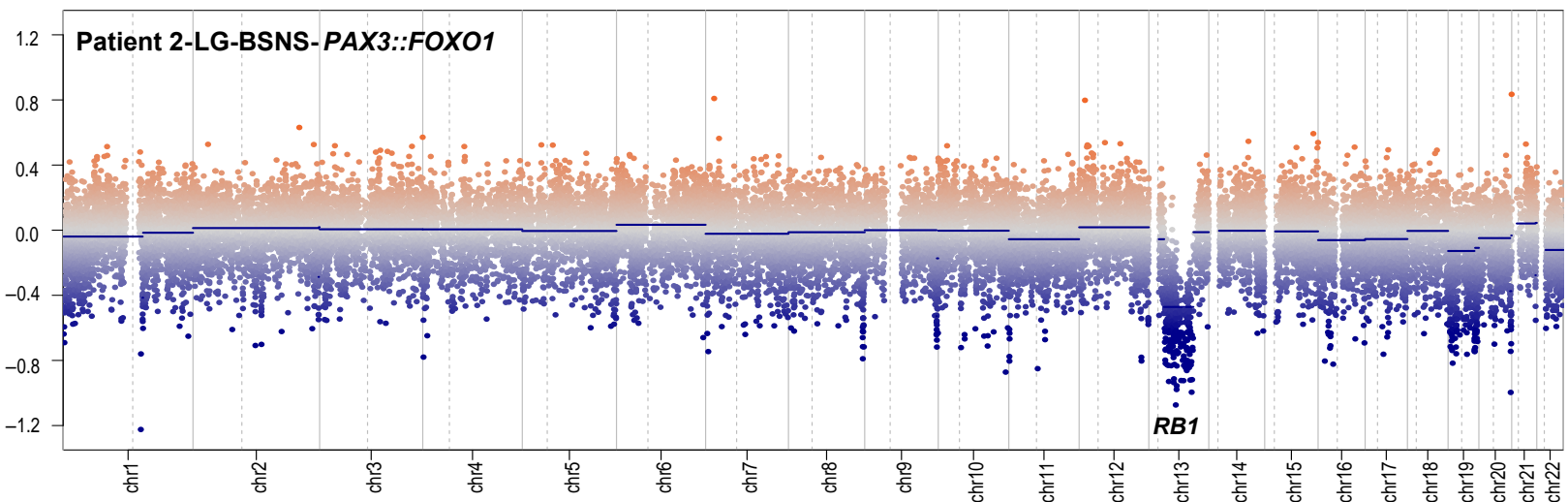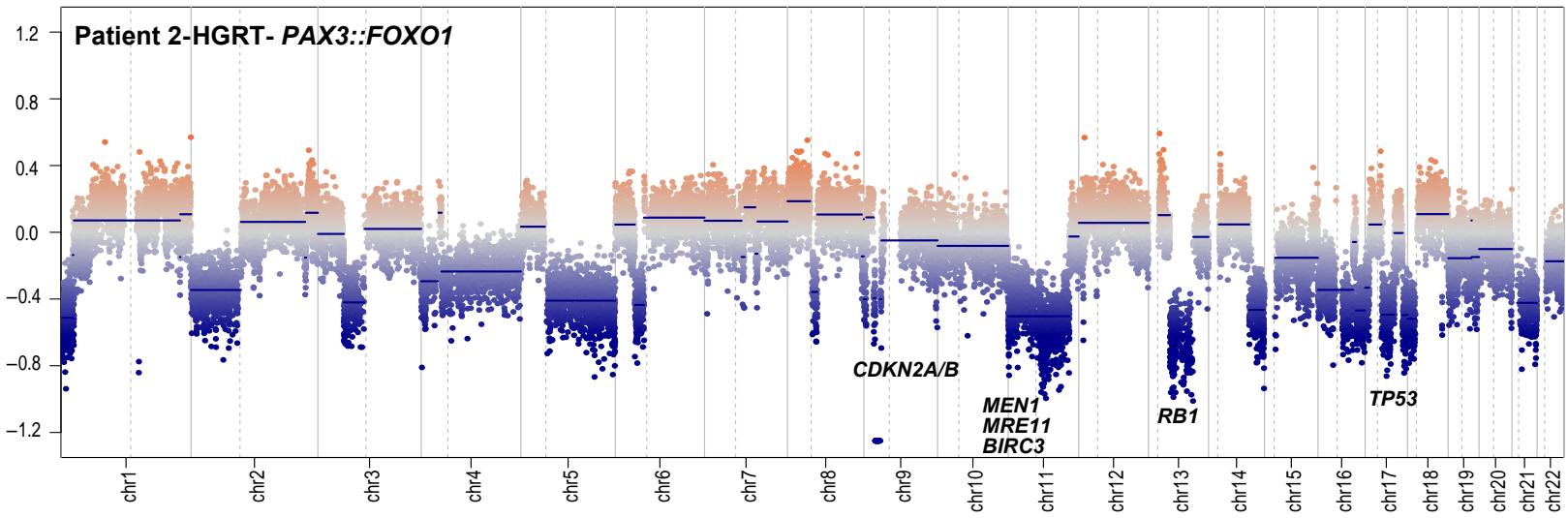

Supplement: Supplementary file 2 — Supplementary file2 (PDF 34913 KB) [file 12105_2026_1923_MOESM2_ESM.pdf]
